# Evaluation of agmatine’s anti-cancer efficacy in Caco-2 colorectal adenocarcinoma cells

**DOI:** 10.1007/s12032-026-03258-x

**Published:** 2026-01-20

**Authors:** Esra Guzel Tanoglu, Muhammed Said Gokce, Miray Karamese, Sevde Altuntas, Ahsen Merve Bayrak, Alpaslan Tanoglu

**Affiliations:** 1 Department of Molecular Biology and Genetics, University of Health Sciences Turkey, Istanbul, 34668 Turkey; 2 Experimental Medicine Research and Application Center, University of Health Sciences Turkey, Istanbul, 34662 Turkey; 3 Department of Tissue Engineering, Institute of Health Sciences, University of Health Sciences Turkey, Istanbul, 34668 Turkey; 4 Department of Internal Medicine, Division of Gastroenterology, Bahçeşehir University, Istanbul, Turkey

**Keywords:** Colorectal cancer, Drug resistance, Agmatine, ABC genes, Therapeutic agent

## Abstract

This study aimed to evaluate the potential effects of agmatine on cell viability, migration, invasion, apoptosis, and the expression of the *ABCB1*,* ABCC1*, and *ABCG2* genes in the Caco-2 colon cancer cell line. Agmatine efficacy was assessed thruogh proliferation, migration, and invasion assays at various concentrations. The apoptotic index was determined using apoptosis-related markers (*Bax*,* Bcl-2*, *Csp-3*) via apoptosis assays, quantitative real-time PCR (qRT-PCR), and Western blot analysis. Expression levels of the *ABCG2*,* ABCB1*, and *ABCC1* genes were measured by qRT-PCR in agmatine-treated Caco-2 cells. Oxidative stress markers, including glutathione peroxidase (*GPx*) and catalase (*CAT*), were evaluated by qRT-PCR. Cell viability analysis revealed that agmatine exerted its most pronounced effects at 72 h, with significant reductions at concentrations of 6, 7.3, and 9 mM in Caco-2 cells and 6, 6.25, and 9 mM in L929 cells (*p* < 0.05). At these concentrations, migration and invasion assays showed dose-dependent decreases in cell motility and invasiveness in Caco-2 cells. Apoptosis analysis demonstrated a significant increase in the apoptotic index with rising agmatine concentrations. Significant decreases in *GPx* and *CAT* were observed in all three agmatine-treated Caco-2 groups compared to untreated controls (*p* < 0.01). However, the expression levels of *ABCG2*, *ABCB1*, and *ABCC1* showed no significant changes following agmatine treatment (*p* > 0.05). These findings indicate that agmatine exerts antiproliferative, anti-migratory, anti-invasive, and pro-apoptotic effects in Caco-2 colon cancer cells, potentially through the modulation of apoptosis- and oxidative stress–related pathways. The lack of significant impacts on *ABC* transporter gene expression suggests that agmatine may be a promising candidate molecule for further translational studies in colorectal cancer.

## Introduction

Worldwide, the incidence of colorectal cancer (CRC) is increasing each year, and is becoming a significant public health concern [1]. Currently, chemotherapy protocols based on the combination of fluorouracil (5-FU) and oxaliplatin (OXA) constitute one of the backbones of CRC treatment. Nevertheless, because currently used therapeutic agents also penetrate healthy tissues, patients may experience many kinds of side effects [2]. Yet, all these treatment options may not be equally effective for every patient and can entail serious side effects as well as resistance-related challenges [3]. Consequently, it is of great importance to explore new therapeutic and supportive agents for CRC therapy.

Polyamines can interact with DNA, RNA, ATP, proteins, and phospholipids under physiological conditions [4]. Polyamine synthesis is essential for sustaining the continuous proliferation characteristic of cancer cells. Notably, the genes encoding two rate limiting enzymes in polyamine biosynthesis, ornithine decarboxylase (ODC; encoded by ODC1) is often dysregulated in various cancers [5].

Agmatine has been reported to confer protective effects against ischemic injury and chronic neuropathic pain in mammals [6]. It has also been identified as having neuroprotective and antioxidant properties in pathophysiological conditions such as anxiety disorders and depression [7, 8]. In contrast to the proliferative effects of the aforementioned polyamines, the polyamine agmatine has been shown to reduce ODC activity and the intracellular uptake of polyamines [9].

Furthermore, emerging evidence suggests that agmatine exerts antiproliferative and pro-apoptotic effects in various cancer models such as prostate hepatocellular carcinoma and glioma primarily by interfering with polyamine metabolism and modulating key signaling pathways [9–11]. In colorectal cells, agmatine has been shown to inhibit cell proliferation and induce cell cycle arrest, potentially through the suppression of ODC activity and the disruption of polyamine homeostasis [11, 12]. These findings highlight agmatine’s potential to serve as a selective therapeutic against in cancer, particularly in tumors with high polyamine requirements, while sparing normal cells in which it exhibits protective and antioxidant properties [13]. Collectively, these findings position agmatine as a promising therapeutic candidate for colorectal cancer, warranting further investigation into its clinical potential [14].

One of the principal mechanisms underlying resistance to anticancer drugs is multidrug resistance (MDR), in which cancer cells overexpress the ATP-binding cassette (ABC) transporter proteins that actively secrete various chemotherapeutic agents from the cell [15]. In particular, the transporters encoded by *ABCB1 (MDR1/P-gp)*,* ABCC1 (MRP1)*, and *ABCG2 (BCRP)* play crucial roles in pumping numerous chemotherapy drugs out of the cell, thus limiting intracellular drug accumulation and enabling tumor cell survival [16, 17].

This study aimed to examine whether agmatine has potential regulatory effects on cell viability, migration and invasion, apoptosis, reactive oxygen species (ROS), and the gene expression levels of *ABCB1*,* ABCC1*, and *ABCG2* transporters, which play important roles in drug resistance in the human colon carcinoma cell line Caco-2.

## Materials and methods

### Cell culture

The Caco-2 and L929 cell lines were obtained from the American Type Culture Collection (Manassas, VA, USA). Caco-2 cells were cultured in high-glucose DMEM (EcoTechBio, Türkiye) mixed with 10% fetal bovine serum (FBS) Capricorn (Ebsdorfergrund, Germany) and 1% penicillin/streptomycin (Gibco Waltham, USA) at 37 °C and 5% CO₂. All experiments were performed using cells at passages 17–27. Experiments were performed in triplicate. Untreated Caco-2 cells were used as the control group in all experiments.

### MTT analysis

Caco-2 and L929 cells were seeded in 96-well plates (20 × 10³ cells/well, 100 µl medium) and incubated for 24 h. Caco-2 cells were treated with agmatine at concentrations of 1, 3, 6, 9, 12, 15, 18, and 21 mM, while L929 cells were treated with 6, 9, 12, 15, 18, and 21 mM for 24, 48, and 72 h. Cell viability was assessed using the MTT assay. Briefly, 5 mg/mL MTT solution was added to the cells and incubated for 4 h. The medium was then removed, and 100 µL of DMSO was added to each well and incubated for 30 min. Absorbance was measured at 570 nm. All experiments were performed in triplicate.

### Migration and invasion assay

Transwell assays were performed using 8 μm pore inserts (Corning, NY, USA). For migration, 2 × 10⁴ Caco-2 cells were seeded in the upper chamber with serum-free medium and treated with agmatine (6, 7.3, and 9 mM) for 72 h, while 750 µl supplemented medium was added to the lower chamber. Non-migrated cells were removed, and migrated cells were fixed (4% paraformaldehyde, 100% methanol), stained with 0.1% crystal violet, and counted in three random fields under an inverted microscope. For invasion, 2 × 10⁴ cells were seeded in Matrigel-coated upper chambers, incubated for 24 h, and then treated with agmatine (6, 7.3, and 9 mM) for 72 h. The lower chamber contained medium with 10% FBS as a chemoattractant. Non-invasive cells were removed, and invasive cells were fixed, stained, and quantified as above.

### Apoptosis assay

Caco-2 and L929 cells were seeded in 96-well plates (2 × 10⁴ cells/well) and incubated for 24 h. After PBS wash, Caco-2 cells were treated with agmatine (6, 7.3, and 9 mM) and L929 (6 mM, 6.25 mM, and 9 mM) for 72 h. Cells were then stained with DAPI (1.8 µl, 1.11 mg/ml) and propidium iodide (PI;1.17 µl, 0.85 mg/ml) for 25 min at 37 °C in the dark, washed with PBS, and analyzed for apoptosis using an fluorescence microscope.

### Ninhydrin analysis

Caco-2 cells (2 × 10⁵/well) were seeded in 6-well plates and incubated for 24 h, then treated with agmatine (6, 7.3, and 9 mM) for 72 h. Cells were harvested with 0.25% trypsin-EDTA, pelleted (300 rpm, 5 min), resuspended in 5% TCA, and incubated at 4 °C for 15 min. After centrifugation (12,000×g, 10 min, 4 °C), supernatants were collected. For the assay, aliquots were mixed with 2% ninhydrin (Bio Basic Inc., Canada) and heated at 100 °C for 5 min, and absorbance was measured at 440 nm.

### RNA isolation and cDNA synthesis

Total RNA was extracted using Trizol reagent (BioBasic, Markham, Canada) according to the manufacturer’s instructions. The concentration and purity of RNAs were determined with spectrophotometry using NanoDrop (Thermo Fisher Scientific, Maryland, USA). cDNA synthesis was conducted using the OneScript^®^ Plus cDNA Synthesis Kit (Applied Biological Materials Inc., Richmond, BC, Canada) according to the manufacturer’s instructions.

### Real-time qPCR

Quantitative real-time PCR (qRT-PCR) was performed using the BlasTaq™ 2X qPCR Master Mix Kit (Applied Biological Materials Inc., BC, Canada) according to the manufacturer’s instructions. The relative expression levels of *ABCC1*,* ABCB1*,* ABCG2*, *Bax*,* Bcl-2*,* Caspase-3 (Csp-3)*, glutathione peroxidase (*GPx*) and catalase (*CAT*), with beta-actin (SenteBioLab, Ankara, Türkiye) as an internal control, were evaluated with qRT-PCR in a T100™ Thermal Cycler (Bio-Rad, USA).

### Western blot analysis

Caco2 and L929 cell lines were seeded with a density of 3 × 10^5^ cells/well in a 6-well plate. The following day, Caco2 cells were treated with 6 mM, 7.3 mM, and 9 mM agmatine, and L929 cells were treated with 6 mM, 6.25 mM, 9 mM agmatine. After 72 h, whole-cell lysates were prepared using the Mammalian Total Protein Extraction Kit (SERVA, Germany), and protein concentrations were determined using the BCA Protein Assay Macro Kit (SERVA, Germany) according to the manufacturer’s instructions. Thirty µg proteins were run on 12% SDS-PAGE and transferred by using the semi-dry system to a 0.22-µm PVDF membrane. Membranes were blocked in 5% non-fat milk for 1 h and incubated with primer antibodies against Bcl-2 (CST, USA), BAX (CST, USA), cleaved *Csp-3* (CST, USA) and GAPDH (AbClonal, USA) overnight at 4 °C. The following day, membranes were washed three times with 1X TBS-T and probed with HRP-conjugated secondary antibodies (Anti-Rabbit, IgG, AbClonal, USA) for 2 h at room temperature. After incubation, membranes were washed three times with 1X TBS-T and protein bands were detected with Clarity ECL substrate (Bio-Rad, USA) using the Chemi-Doc MP Imaging System (Bio-Rad, USA). Signals were normalized to housekeeping GAPDH, and intensities were calculated using Image Lab Software (version 6.1).

### Statistical analysis

All performed experiments were repeated three times, and the graphical presentations were performed using GraphPad Prism 10.0 (San Diego, CA, USA). Data are presented as the means ± standard deviation and were analyzed using SPSS 22.0 software (Chicago, IL, USA). Target gene expression was normalized to the corresponding B-actin value, and ΔCt values were calculated accordingly. The normality of ΔCt distributions was assessed using the Shapiro-Wilk test. As the ΔCt values did not deviate significantly from a normal distribution, parametric statistical tests were applied. Group comparisons were performed using one-way analysis of variance. When a significant overall group effect was detected, post hoc pairwise comparisons were conducted using Tukey’s honestly significant difference test. Statistical analyses were carried out on ΔCt values, whereas relative gene expression levels were calculated using the 2⁻ΔΔCt method for graphical presentation and descriptive purposes. A two-sided p value of < 0.05 was considered statistically significant.

## Results

**Fig. 1:**
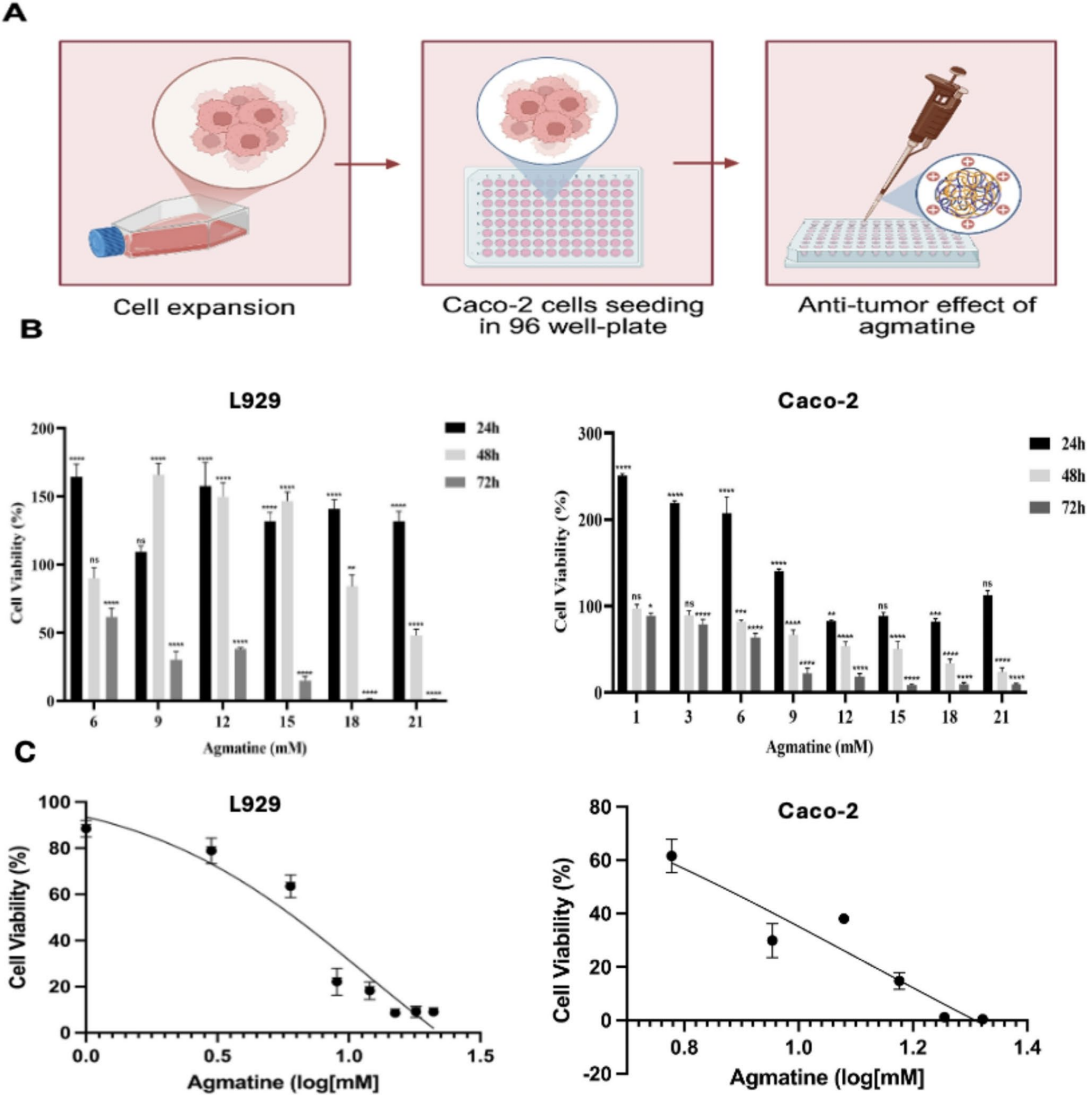
(**A**) Schematic figure of anti-tumor effect of agmatine in Caco-2 cells. (Figure was created using BioRender under an academic license). (**B**) Cell viability (%) of agmatine on the Caco-2 and L929 cells. Caco-2 was treated with different concentrations of agmatine 1, 3, 6, 9, 12, 15, 18, and 21 mM and L929 cells were treated with 6, 9, 12, 15, 18, and 21 mM for 24, 48, and 72 h. (**C**) Logarithmic representation of the MTT assay results for the L929 and Caco-2. Error bars show standard error. * also indicates that statistically significant differences (*p < 0.05, **p < 0.01, ***p < 0.001)

**Fig. 2:**
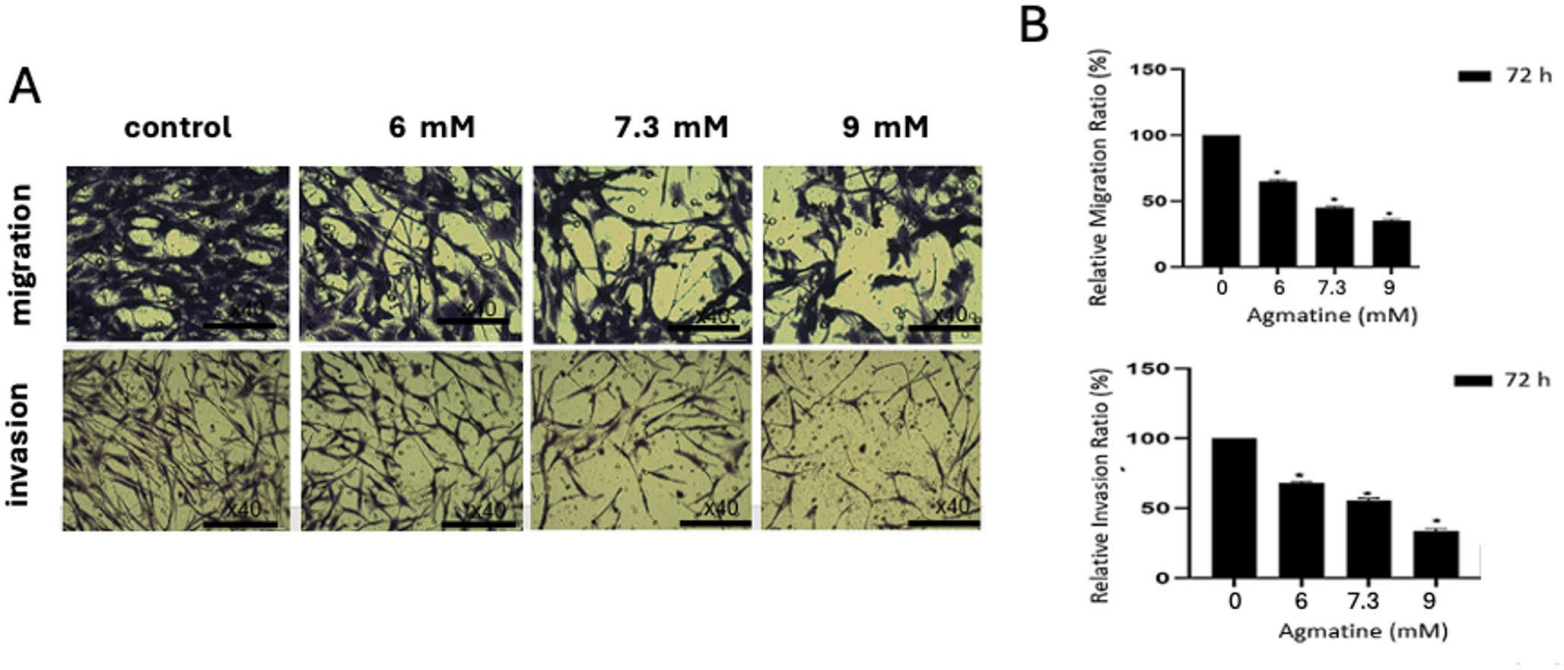
Agmatine inhibited invasion and migration of Caco-2 cells. (**A**)After 72 h of agmatine treatment, the effect on the migration ability of Caco-2 cells was evaluated by transwell migration assay. (**B**) The relative migration ratios of migration cells. (**A**) After 72 h of agmatine treatment, the effect on the invasion ability of Caco-2 cells was evaluated by transwell invasion assay. (**B**) The relative invasion ratios of migration cells. Data were obtained from triplicate experiments and expressed as the means ± standard deviation. *p < 0.05

**Fig. 3:**
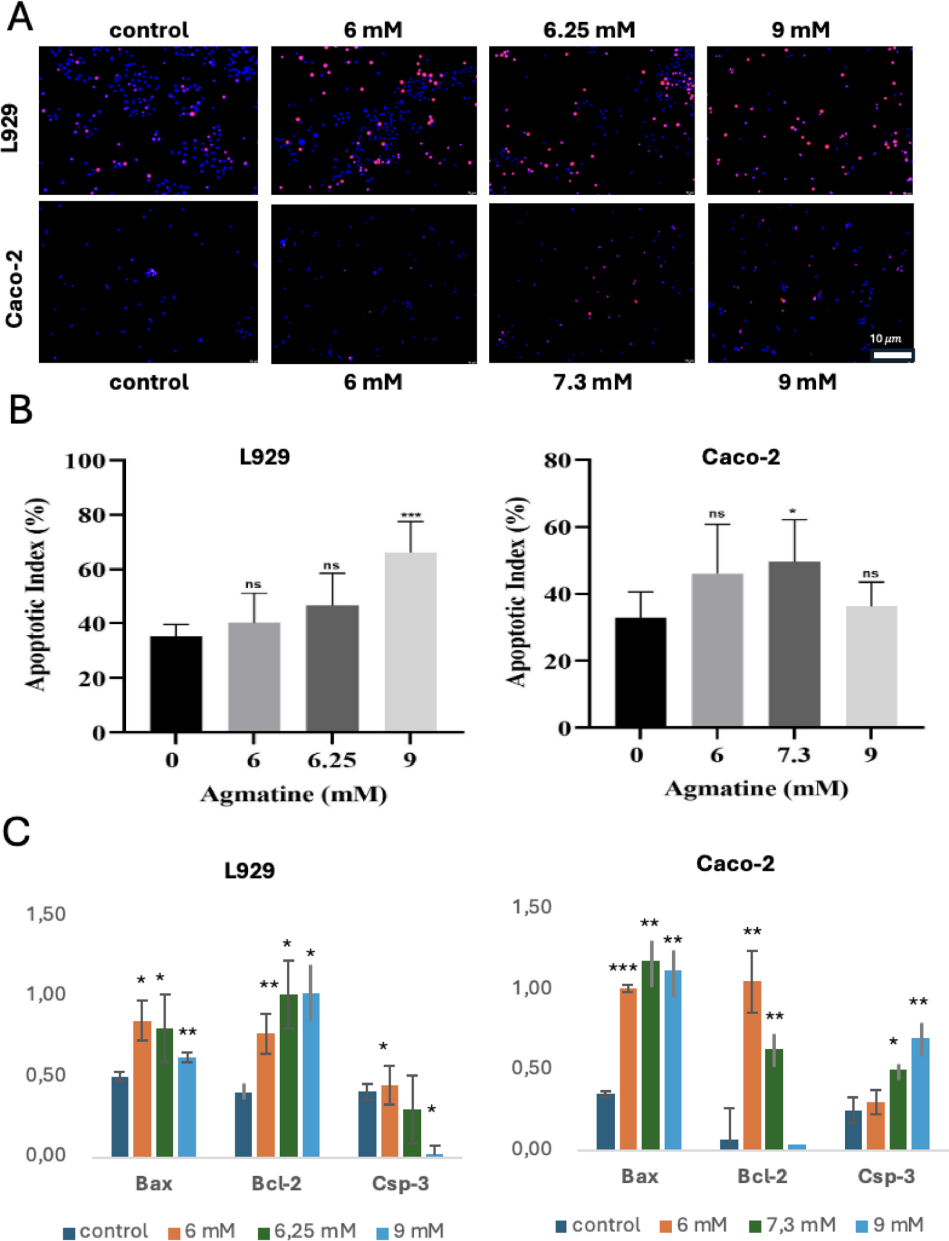
Agmatine induced apoptosis in Caco-2 and L929 cells. (**A**) The apoptosis of Caco-2 cells (6, 7.3, and 9 mM) and L929 (6, 6.25 and 9 mM) after agmatine treatment was observed by DAPI and PI staining for 72 h. (**B**) The apoptotic index (%) values of Caco-2 and L929 cells. (**C**) Relative expression levels of Bax, Bcl-2 and Csp-3 in agmatine-treated Caco-2 and L929 cells versus control groups. Data were obtained from triplicate experiments and expressed as the means ± standard deviation. *p < 0.05, **p < 0.01, ***p < 0.001

A schematic representation of the anti-tumor effects of agmatine in Caco-2 cells is illustrated in Fig. 1A. As shown in Fig. 1B, the viability of both Caco-2 and L929 cells decreased in a concentration-dependent manner with increasing agmatine concentrations. The half-maximal inhibitory concentration (IC₅₀) of agmatine at 72 h were determined to be 7.3 mM for Caco-2 cells and 6.25 mM for L929 cells. Overall, a significant increase in inhibition rate was observed in Caco-2 cells following agmatine treatment. Therefore, agmatine doses corresponding to concentrations below and above the IC₅₀ values (6, 7.3, and 9 mM for Caco-2 cells; 6, 6.25, and 9 mM for L929 cells) were selected for subsequent experiments. Fig. 1C presents the logarithmic representation of the MTT assay results for the cells.The effects of agmatine on the migration and invasion of Caco-2 cells were evaluated using transwell migration and Matrigel invasion assays. As shown in Fig. 2A the number of Caco-2 cells migrating through the membrane into the lower chamber was significantly and dose-dependently reduced in the agmatine-treated groups compared with control groups. When Caco-2 cells were treated with agmatine at concentrations of 6, 7.3, and 9 mM for 72 h, their relative migration rates decreased from 61.2% to 46.3% and further to 30.02%, respectively (Fig. 2A; *p* < 0.05). Similarly, agmatine was observed to inhibit Caco-2 cell invasion in a dose-dependent manner. At 72 h, treatment with 6, 7.3, and 9 mM agmatine resulted in a reduction of the relative invasion rate from 54.6% to 33.4% and further to 18.8%, respectively (Fig. 2B; *p* < 0.05). Collectively, both assays demonstrated that the numbers of migrated and invaded Caco-2 cells were significantly decreased in a dose-dependent manner in agmatine-treated groups (6, 7.3, and 9 mM) compared with untreated Caco-2 cells (control). The effects of agmatine on the apoptosis of Caco-2 and L929 cells were evaluated by fluorescence microscopy using DAPI and PI staining. DAPI/PI staining revealed that after agmatine treatment with 6, 7.3, or 9 mM in Caco-2 and 6, 6.25, or 9 mM in L929 for 72 h, apoptosis rates were increased dose-dependently when compared to the control cells (Fig. 3A). In Caco-2 cells treated with 6, 7.3, or 9 mM agmatine for 72 h, the apoptotic indices were 46% (ns), 50% (*p* < 0.05), and 36% (ns), respectively, compared to the untreated control. In contrast, agmatine treatment in L929 cells induced a dose-dependent increase in the apoptotic index, reaching 40% (ns), 46% (ns), and 66% (*p* < 0.001) at 6, 6.25, and 9 mM concentrations, respectively (Fig. 3B).

The expression levels of the apoptosis-related genes, *Bax*,* Bcl-2* and *Casp-3* were evaluated in Caco-2 cells across the control, 6 mM, 7.3 mM, and 9 mM groups using ΔCt values normalized to *B-actin* (Fig. 3C). For *Bax*, the mean ΔCt value in the control group was 0.35 ± 0.02, compared with 1.01 ± 0.19 in the 6 mM group, 1.18 ± 0.07 in the 7.3 mM group, and 1.12 ± 0.13 in the 9 mM group, indicating a significant increased difference in the 6 mM, 7.3 mM, and 9 mM groups compared to the control (*p* < 0.001, *p* < 0.01, *p* < 0.01).

When the 6 mM groups were compared to the control group, the expression levels of *Bcl-2* increased in Caco-2. However, expression in the 7.3 and 9 mM groups decreased relative to the 6 mM group cells. The overall difference between groups was statistically significant without 9 mM, compared with the control (*p* < 0.01, *p* < 0.01, ns).

The mean ΔCt value of *Csp-3* was increased in the 7.3 mM, and 9 mM groups compared to the control group, with a significant group effect (*p* < 0.05, *p* < 0.001).

qRT-PCR analysis revealed dose-dependent upregulation of *Bcl-2* and downregulation of *Bax* and *Csp-3* expression in agmatine-treated (6, 6.25, 9 mM) L929 cells, resulting in significant differences relative to the control (Fig. 3C).

**Fig. 4:**
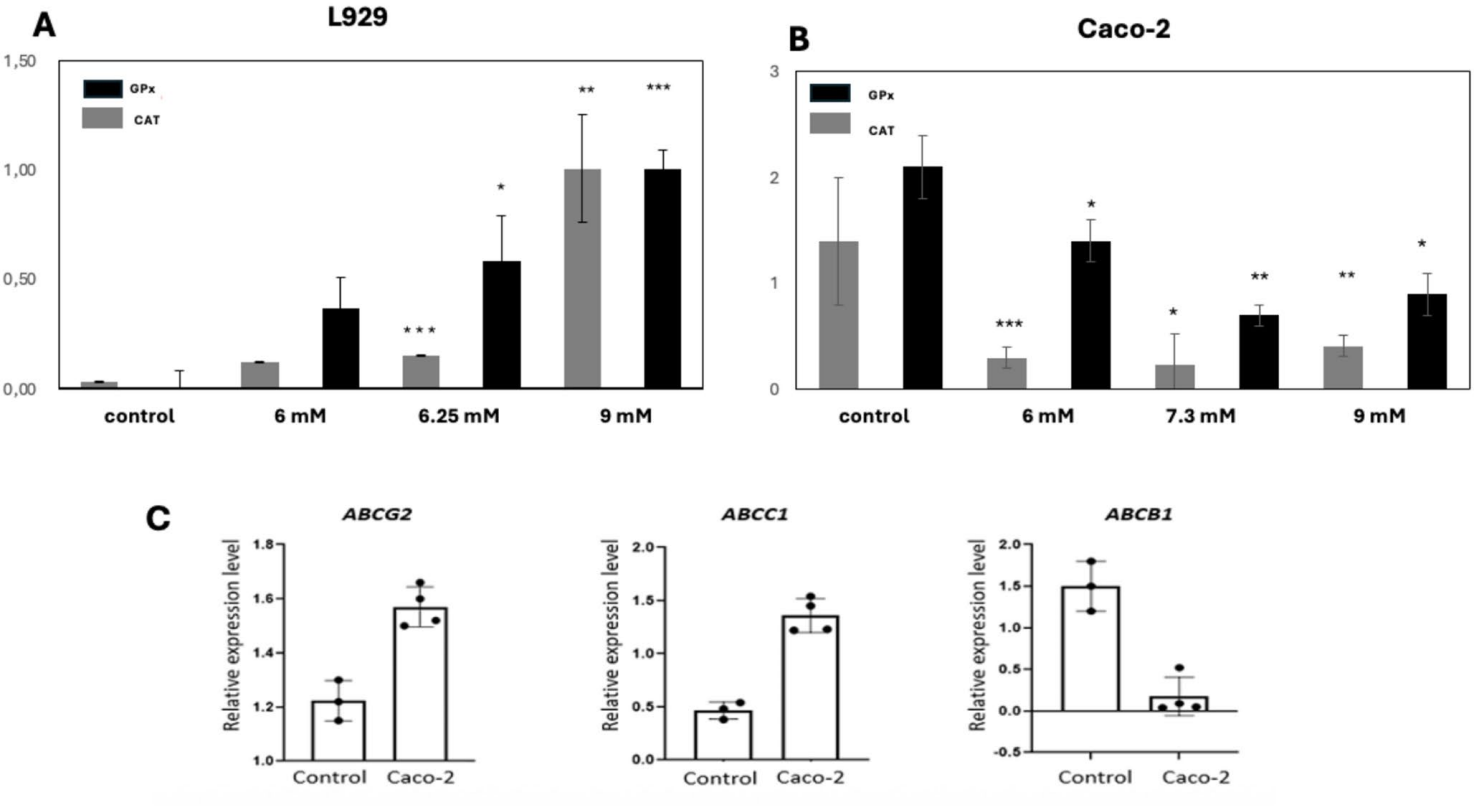
Relative Expression Levels of GPx, CAT, ABCG2, ABCC1, and ABCB1 in L929 and Caco-2 Cells Treated with Agmatine. (**A**) The expression levels of GPx and CAT in L929 cells were modulated by agmatine in a dose-dependent manner (**B**) The expression levels of GPx and CAT in Caco-2 cells were modulated by agmatine in a dose-dependent manner C) Relative expression levels of ABCG2, ABCC1, and ABCB1 in Caco-2 cells treated with 7.3 mM agmatine for 72 h compared to control. Data were obtained from triplicate experiments and expressed as the means ± standard deviation. *p < 0.05, **p < 0.01, ***p < 0.001

To investigate the effects of agmatine-induced ROS formation in Caco-2 and L929 cells, GPx and catalase (*CAT*) mRNA levels were assessed using qRT-PCR. In contrast to the control group, a significant decreases in *GPx* and *CAT* expression were observed in all three agmatine-treated groups of Caco-2 cells compared to the untreated controls. In L929 cells, however, *GPx* and *CAT* mRNA levels increased in a dose-dependent (6.25 and 9 mM) and linear manner relative to the control group (Fig. 4A and B).

The relative expression levels of the *ABCG2*, *ABCC1*, and *ABCB1* genes were evaluated with qRT-PCR after 7.3 mM agmatine treatment of Caco-2 cells. qRT-PCR data revealed no significant differences between the *ABCG2*, *ABCC1*, and *ABCB1* expression levels and agmatine-treated Caco-2 cells (*p* > 0.05; Fig. 4C).

Western blot analyses performed to assess apoptotic pathways (*Bax*,* Bcl-2*, and *Csp-3*) in Caco-2 cells treated with agmatine at concentrations of 6 mM, 7.3 mM, and 9 mM revealed no detectable bands for these proteins, despite showing clear and consistent GAPDH bands as the internal loading control (data not shown). The absence of detectable bands may indicate that agmatine does not strongly activate the apoptotic pathway at the protein level in this cell model or that the experimental conditions (e.g., insufficient lysate loading or low basal expression of these apoptotic proteins) were inadequate to yield a visible signal on the blot.

## Discussion

To the best of our knowledge, the present study is the first to demonstrate the anti-proliferative, anti-migration, anti-invasive, and anti-apoptotic effects of agmatine in the Caco-2, a model widely used for its enterocyte-like differentiation properties [18].

Colon epithelial cells are exposed to numerous dietary compounds and metabolites that can affect cell physiology. Among these, polyamines are found in plant- and animal-based foods and fermented food products [19]. Polyamines are also produced by luminal colon bacteria. It has been reported that agmatine reduces cell proliferation in the HT-29 cell lineand that treating HT-29 cells with agmatine at a concentration of 1 mM reduces cell proliferation and leads to complete inhibition at a dose concentration of 5 mM [20].

In the literature, it has been shown that agmatine affects ADC-dependent cell apoptosis in the CRC cell line (HCT116) [21]. Agmatine produces a concentration-dependent inhibition of proliferation in six colorectal cancer cell lines, while colon carcinoma tissue samples displayed significantly lower agmatine levels compared to normal tissue [11]. Furthermore, agmatine demonstrated antiproliferative effects in tumor cells derived from colonic, hepatic, and neuronal origins [12]. Plasma agmatine levels in prostate cancer patients have been found to be significantly lower compared to healthy individuals; agmatine inhibits proliferation in prostate cancer cells and has been proposed as a potential biomarker and therapeutic agent [9]. In another study, agmatine produced a dose-dependent antiproliferative effect in MCF-7 breast cancer cells and SK-MG-1 glioma cells, while also suppressing polyamine uptake [10, 22].

Our results demonstrate that agmatine at concentrations of 6 mM, 7.3 mM, and 9 mM significantly inhibits ROS formation in Caco-2 cells at 72 h, suggesting that agmatine possesses the ability to alleviate oxidative stress and prevent ROS-induced oxidative damage.

Agmatine has been shown to reduce ROS production by activating the Nrf2/HO-1 and PI3K/Akt pathways in LPS-induced BV-2 microglia and RAW 264.7 macrophage cells, while increasing GPx activity and glutathione levels and inducing a “pre-adaptive response” [23]. In a diabetes mellitus model, agmatine treatment has been demonstrated to elevate GPx and catalase levels in leukocytes and to reduce oxidative/nitrosative stress [24]. Additionally, agmatine has been reported to inhibit tumor cell proliferation by modulating polyamine metabolism in HT-29 cells, with agmatine levels being significantly lower in colon cancer tissues compared to normal tissue, suggesting a potential association with polyamine dysregulation [11]. Another study by Park et al. examined the protective effects of agmatine against cisplatin-induced cell death in an auditory cell line. The results indicated that agmatine inhibits apoptosis by suppressing *Bax* and *Csp-3* expression, though it does not directly decrease ROS levels [25]. These protective effects are thought to be associated with the regulatory role of agmatine on oxidative stress and inflammation [26].

To the best of our knowledge, this is the first study to evaluate ABC genes in agmatine treated Caco-2 cancer lines. A previous study has investigated the relationships between *ABCG2*, *ABCC1*, and *ABCB1* genes in two different colon cancer cell lines (Caco-2 and HT-29) [27]. In the HT-29 cell line, a significant difference was found among the *ABCB1*, *ABCG2*, and *ABCC1* genes. It was shown that there was high up regulation of *ABCB1* and *ABCG2* but low upregulation of the *ABCC1* compared to the other genes. In the Caco-2 cell line, there was shown to be significant difference in all three genes; as a result, suggesting that the upregulation of expression of ABC genes is directly related to cancer cell line [27]. *ABCB1*,* ABCC1*, and *ABCC2* mRNA levels were compared between Caco-2 cells and other colorectal cancer cell lines (HCT-15, LoVo, DLD-1, HCT-116, SW620). The results showed that *ABCB1* and *ABCC2* expression were higher in Caco-2 cells, whereas no significant differences were observed in HT-29-like cell lines [28]. In another study that included the Caco-2 and HT-29 cell lines, the promoter methylation levels of *ABCB1*, *ABCC1*, and *ABCG2* were investigated. The *ABCC1* promoter exhibited low methylation in both cell lines, while differences were observed in the methylation patterns of *ABCB1* and *ABCG2* [29]. According to our current results, no significant difference was found in the Caco-2 cell line that was treated with agmatine. Our findings indicate that the major ABC transporters *ABCG2*, *ABCC1*, and *ABCB1* are not modulated by agmatine treatment in colon cancer cells and do not mediate resistance to agmatine in Caco-2 tumor cells.

According to the mRNA expression levels of *ABCB1*,* ABCC1*, and *ABCG2* transporters in colorectal cancer patients, there is a significant difference in the expression levels of these ABC transporters. The expression levels were found to be significantly higher in cancer patients. These findings showed that the expression of ABC transporters is a potential biomarker for the diagnosis of CRC patients [30, 31].

## Limitations of the study

This study included no vehicle control or known cytotoxic positive control agents, such as cisplatin or doxorubicin. In this context, the absence of a positive cytotoxic control limits the direct comparison of our findings with the standard chemotherapeutic agents currently used in clinical practice. However, the primary objective of this study was to investigate the intrinsic biological effects of agmatine on CRC cells. In this study, the inclusion of only the Caco-2 cell line among CRC cell lines. On the other hand, the inclusion of only the Caco-2 cell line, without testing other colon cancer cell lines, represents one of the limitations of this current work.

The Caco-2 cell line was selected because it is a widely used and well-characterized in vitro model in CRC research, and it is particularly suitable for investigating cellular processes such as proliferation, migration, and invasion. However, the inclusion of additional colorectal cancer cell lines with distinct genetic and molecular characteristics would have enhanced the translational relevance of the findings.

One other limitation of the present study is that Western blotting failed to detect bands corresponding to *Bax*,* Bcl-2*, and *Csp-3* in agmatine-treated Caco-2 cells, despite successfully visualizing of the internal control GAPDH. Consequently, changes in the expression of these proteins could not be confirmed at the protein level, limiting the validation of observations potentially made at the transcriptional or other levels.

## Conclusion

These findings highlight agmatine as a promising candidate for further investigation in colorectal cancer therapy, particularly through the modulation of proliferation, invasion, apoptosis, and oxidative stress pathways. The lack of effects on ABC transporters further supports its potential as a selective and non-MDR-related agent. Future studies including additional CRC cell lines, in vivo models, and comparisons with established chemotherapeutic agents will be essential for fully elucidating its therapeutic potential and mechanisms of action.

## Data Availability

Data will be made available on request.
